# *KIT* D816 mutated/CBF-negative acute myeloid leukemia: a poor-risk subtype associated with systemic mastocytosis

**DOI:** 10.1038/s41375-018-0346-z

**Published:** 2019-01-11

**Authors:** Mohamad Jawhar, Konstanze Döhner, Sebastian Kreil, Juliana Schwaab, Khalid Shoumariyeh, Manja Meggendorfer, Lambert L. F. Span, Stephan Fuhrmann, Nicole Naumann, Hans-Peter Horny, Karl Sotlar, Boris Kubuschok, Nikolas von Bubnoff, Karsten Spiekermann, Michael Heuser, Georgia Metzgeroth, Alice Fabarius, Stefan Klein, Wolf-Karsten Hofmann, Hanneke C. Kluin-Nelemans, Torsten Haferlach, Hartmut Döhner, Nicholas C. P. Cross, Wolfgang R. Sperr, Peter Valent, Andreas Reiter

**Affiliations:** 1 Department of Hematology and Oncology, University Hospital Mannheim, Heidelberg University, Mannheim, Germany; 2 Department of Internal Medicine III, University Hospital Ulm, Ulm, Germany; 3 Department of Hematology, Oncology and Stem Cell Transplantation, Medical Center, Faculty of Medicine, University of Freiburg, Freiburg, Germany; 4 German Cancer Consortium (DKTK) Partner Site Freiburg, Freiburg, Germany; 5 Munich Leukemia Laboratory, Munich, Germany; 6 Department of Hematology, University Medical Center Groningen, University of Groningen, Groningen, The Netherlands; 7 Department of Hematology and Oncology, HELIOS Hospital, Berlin, Germany; 8 Institute of Pathology, Ludwig-Maximilians-University, Munich, Germany; 9 Institute of Pathology, Medical University of Salzburg, Salzburg, Austria; 10 Department of Internal Medicine I, José-Carreras Centrum for Immuno- and Gene Therapy, University of Saarland Medical School, Homburg/Saar, Germany; 11 Department of Medicine III, University Hospital of Munich, Munich, Germany; 12 Department of Hematology, Hemostasis, Oncology and Stem Cell Transplantation, Hannover Medical School, Hannover, Germany; 13 Wessex Regional Genetics Laboratory, Salisbury, UK; 14 Faculty of Medicine, University of Southampton, Southampton, UK; 15 Department of Internal Medicine I, Division of Hematology and Ludwig Boltzmann Cluster Oncology, Medical University of Vienna, Vienna, Austria

**Keywords:** Acute myeloid leukaemia, Myeloproliferative disease

## Abstract

*KIT* D816 mutations (*KIT* D816^mut^) are strongly associated with systemic mastocytosis (SM) but are also detectable in acute myeloid leukemia (AML), where they represent an adverse prognostic factor in combination with core binding factor (CBF) fusion genes. Here, we evaluated the clinical and molecular features of *KIT* D816^mut^/CBF-negative (CBF^neg^) AML, a previously uncharacterized combination. All *KIT* D816^mut^/CBF^neg^ cases (*n* = 40) had histologically proven SM with associated AML (SM-AML). Molecular analyses revealed at least one additional somatic mutation (median, *n* = 3) beside *KIT* D816 (e.g., *SRSF2*, 38%; *ASXL1*, 31%; *RUNX1*, 34%) in 32/32 (100%) patients. Secondary AML evolved in 29/40 (73%) patients from SM ± associated myeloid neoplasm. Longitudinal molecular and cytogenetic analyses revealed the acquisition of new mutations and/or karyotype evolution in 15/16 (94%) patients at the time of SM-AML. Median overall survival (OS) was 5.4 months. A screen of two independent AML databases (AML^databases^) revealed remarkable similarities between *KIT* D816^mut^/CBF^neg^ SM-AML and *KIT* D816^mut^/CBF^neg^ AML^databases^ (*n* = 69) with regard to *KIT* D816^mut^ variant allele frequency, mutation profile, aberrant karyotype, and OS suggesting underlying SM in a significant proportion of AML^databases^ patients. Bone marrow histology and reclassification as SM-AML has important clinical implications regarding prognosis and potential inclusion of KIT inhibitors in treatment concepts.

## Introduction

According to the World Health Organization (WHO) classification, advanced systemic mastocytosis (advSM) comprises aggressive SM, SM with an associated hematologic neoplasm (SM-AHN), and mast cell leukemia [1–3]. SM-AHN is the most frequent subtype diagnosed in up to 80% of advSM patients [4]. The AHN is characterized in >90% of patients as a myeloid neoplasm, e.g., myelodysplastic/myeloproliferative neoplasm unclassifiable (SM-MDS/MPN-u), chronic myelomonocytic leukemia (SM-CMML), myeloproliferative neoplasm (SM-MPN), myelodysplastic syndrome (SM-MDS), or acute myeloid leukemia (SM-AML) [4].

In general, acquired mutations in *KIT* (usually *KIT* D816V) are detectable in >90% of patients with SM, acknowledged to be most relevant for disease pathogenesis [5]. In advSM, multi-lineage involvement (including non-mast-cell-lineage cells, e.g., monocytes, eosinophils, and others) of *KIT* mutations is frequently observed and the basis for the phenotype of SM-AHN [6–8]. Recent data have, however, also highlighted that the molecular pathogenesis of advSM is much more complex with the presence of one or more additional somatic mutations, e.g., in *SRSF2*, *ASXL1*, *RUNX1*, *JAK2*, *TET2* [9–11]. These additional mutations are often acquired by neoplastic (stem) cells prior to *KIT* D816V thereby indicating a multi-mutated stem cell disease and a step-wise process of oncogenesis [12].

Core binding factor (CBF) positive AML (CBF^pos^ AML) represents 5–8% of all AMLs and is defined by the presence of a t(8;21)(q22;q22) and the associated *RUNX1*–*RUNX1T1* fusion gene, or an inv(16)(p13.1q22)/t(16;16)(p13.1;q22) with the resulting *CBFB*–*MYH11* fusion gene. CBF^pos^ AML is categorized to the genetically favorable risk group. However, *KIT*^.^ mutations, most frequently at position D816 (*KIT* D816^mut^), are detectable in up to 45% of CBF^pos^ patients and associated with adverse prognosis [13, 14]. The potential association of *KIT* D816^mut^/CBF^pos^ AML with underlying SM has been described in various case reports, case-series, and/or literature reviews [15–19], however, there is little information available on *KIT* D816^mut^/CBF^neg^ AML [20]. We therefore evaluated (a) clinical and molecular genetic characteristics, (b) response to treatment, and (c) survival and prognostic factors in 40 patients with *KIT* D816^mut^/CBF^neg^ AML collected at 4 centers of the European Competence Network on Mastocytosis (ECNM). To further investigate whether *KIT* D816^mut^/CBF^neg^ defines a distinct AML subtype associated with SM and poor prognosis, two independent AML databases (AML^databases^, German/Austrian AML Study Group, Munich Leukemia Lab) were retrospectively screened for *KIT* D816^mut^/CBF^neg^ AML patients (selection criteria were all AML patients with available status on CBF and *KIT* D816^mut^).

## Methods

### Diagnosis of SM-AML

The diagnosis of SM-AML was established according to the WHO classification [2, 21–23]. Bone marrow biopsies and smears were evaluated by reference pathologists of the ECNM (H-PH and K Sotlar). A total of 48 CBF^neg^ SM-AML patients, diagnosed in 4 ECNM centers between 2003 and 2018, were included in this retrospective analysis. Eight patients negative for *KIT* D816 mutations (*n* = 5) or with unknown *KIT* D816 mutation status (*n* = 3) were excluded. Among all SM-AML patient from the 4 ECNM centers, one patient was *KIT* D816^mut^/CBF^pos^. The study design adhered to the tenets of the Declaration of Helsinki and was approved by the institutional review board of the Medical Faculty of Mannheim, Heidelberg University, as part of the “German Registry on Disorders of Eosinophils and Mast Cells”. All patients gave written informed consent.

### Molecular analyses

Targeted next-generation sequencing (NGS) was either performed by 454 FLX amplicon chemistry (Roche, Penzberg, Germany) or library preparation based on the TruSeq Custom Amplicon Low Input protocol (Illumina, San Diego, CA, USA) and sequencing on the MiSeq instrument (Illumina) to investigate mutation status of *KIT* and the following 32 genes: *ASXL1*, *BCOR*, *CALR*, *CBL*, *CSNK1A1*, *DNMT3A*, *ETNK1*, *ETV6*, *EZH2*, *FLT3*, *GATA1*, *GATA2*, *IDH1*, *IDH2*, *JAK2*, *KRAS*, *MLL*, *MPL*, *NPM1*, *NRAS*, *PHF6*, *PIGA*, *PTPN11*, *RUNX1*, *SETBP1*, *SF3B1*, *SRSF2*, *TET2*, *TP53*, *U2AF1, ZRSR2*, and *WT1* [9].

Subsequent to bcl2fastq and demultiplexing, alignment and variant calling were performed using JSI SeqNext v4.4.0 (JSI Medical Systems, Kippenheim, Germany) software with default parameters. Only basecalls with quality score of 30 or above were considered for further processing. In median ~1800 reads were aligned to the target region. All regions below the minimal coverage of 400 reads were rejected and resequenced for higher depth. Variants were called with a variant allele frequency (VAF) cutoff of 3% and each assessed manually for pathogenicity. Mutation assessment was performed using COSMIC (v78), dbSNP (v150), ClinVar (2018-07), gnomAD (r2.0.2 and dbNSFP v3.5).

Qualitative and quantitative assessments of *KIT* D816V and *KIT* D816V expressed allele burden, respectively, was performed using allele-specific quantitative real-time reverse transcriptase polymerase chain reaction analyses (qRT-PCR) as previously described [24]. Molecular analyses were performed at diagnosis of SM ± AHN and at diagnosis of SM-AML.

### Conventional cytogenetic analysis and fluorescence in situ hybridization

Cytogenetic analyses of at least 20 Giemsa-banded bone marrow metaphases (24 h and/or 48 h culture) was performed and interpreted according to the International System for Human Cytogenetic Nomenclature [25]. If necessary, chromosome banding analysis was combined with fluorescence in situ hybridization according to the manufacturer's instructions (Metasystems, Altlussheim, Germany) [26].

### Statistical analyses

Statistical analyses considered clinical, laboratory, or molecular parameters obtained at the time of diagnosis. Overall survival (OS) analysis was determined as time from date of diagnosis to date of death or last follow up. Pearson correlation analysis was performed for the correlation between two parameters. Differences in the distribution of continuous variables between categories were analyzed by Mann–Whitney test (for comparison of two groups). For categorical variables, Fisher’s exact test was used. OS probabilities were estimated with the Kaplan–Meier method and compared by the log-rank test in univariate analysis. For the estimation of hazard ratios (HRs) and multivariate analysis, the Cox proportional hazard regression model was used. *P*-values < 0.05 (two-sided) were considered significant. There was no adjustment for multiple testing as all analyses were explorative. SPSS version 22.0.0 (IBM Corporation, Armonk, NY, USA) was used for statistical analysis.

## Results

### Clinical and morphological characteristics

The median age of the 40 *KIT* D816^mut^/CBF^neg^ SM-AML patients was 65 years (range 28–83, male 73%). The median percentage of mast cells in bone marrow trephine biopsies was 10% (range 5–65). Blood parameters analyzed in this study included: leukocytes (median 8.7 × 10^9^/L, range 0.5–71.8), hemoglobin (median 8.3 g/dL, range 5.1–14.3; <10 g/dL in 79% of patients), platelets (median 40 × 10^9^/L, range 5–412; <100 × 10^9^/L in 88% of patients), eosinophils (0.2 × 10^9^/L, range 0–16.7; > 1.0 × 10^9^/L in 18% of patients), and monocytes (0.9 × 10^9^/L, range 0.1–23.5; >1.0 × 10^9^/L in 39% of patients). Median serum tryptase level (normal value < 11.4 µg/L) was 92 µg/L (range 13–885; >100 µg/L in 48% of patients). Signs of non-hematologic organ dysfunction included elevated alkaline phosphatase (AP, normal value < 130 U/L; median 145 U/L; range 52–1428; >150 U/L in 50% of patients), splenomegaly (64%), and ascites (25%) (Table 1a).

**Table 1a:** Clinical characteristics and outcome of 40 patients with *KIT* D816^mut^/CBF^neg^ systemic mastocytosis associated with acute myeloid leukemia (SM-AML)

*n*	Variables	
	No. of patients (*n*)	40
	Age in years, median (range)	65 (28–83)
	Males, *n* (%)	29 (73)
**29**	**Diagnosis prior to SM-AML**	
	ISM, *n* (%)	5 (17)
	SM-AHN, *n* (%)	24 (83)
**24**	**AHN-subtypes**	24 (83)
	MDS/MPN-u, *n* (%)	8 (33)
	CMML, *n* (%)	6 (25)
	MDS, *n* (%)	5 (21)
	MPN-eo, *n* (%)	5 (21)
29	Time to progression to SM-AML in months, median (range)	24 (2–116)
	**SM-related findings**	
21	Mast cell infiltration in BM histology, %; median (range)	10 (5–65)
27	Serum tryptase, µg/L; median (range)	92 (13–885)
	>100 µg/L, *n* (%)	13 (48)
32	Alkaline phosphatase, U/L; median (range)	145 (52–1428)
	>150 U/L, *n* (%)	16 (50)
36	Splenomegaly, *n* (%)	23 (64)
36	Ascites, *n* (%)	9 (25)
	**Outcome**	
	Follow-up, months, median (range)	5 (0–91)
	Death, *n* (%)	30 (75)

*AHN* associated hematologic neoplasm, *BM* bone marrow, *MDS/MPN-u* myelodysplastic/myeloproliferative neoplasm unclassifiable, *CMML* chronic myelomonocytic leukemia, *ISM* indolent SM, *MPN-eo* MPN associated with eosinophilia, *n* number

### De novo SM-AML and secondary SM-AML

De novo SM-AML was diagnosed in 11/40 (28%) patients. Secondary SM-AML evolving from indolent SM (*n* = 5) or SM-AHN (*n* = 24) was observed in 29/40 (73%) patients with a median time to progression of 24 months (range 2–116). The 24 patients with AHN were classified as MDS/MPN-u (*n* = 8), CMML (*n* = 6), MDS (*n* = 5), or MPN associated with eosinophilia (MPN-eo) (*n* = 5) (Table 1a). The comparison between de novo and secondary AML revealed that patients with secondary AML were older, had a higher monocyte count, a higher AP level, and a lower serum tryptase level. However, there were no significant differences regarding OS (*P* = 0.2).

### Somatic mutations

**Fig. 1:**
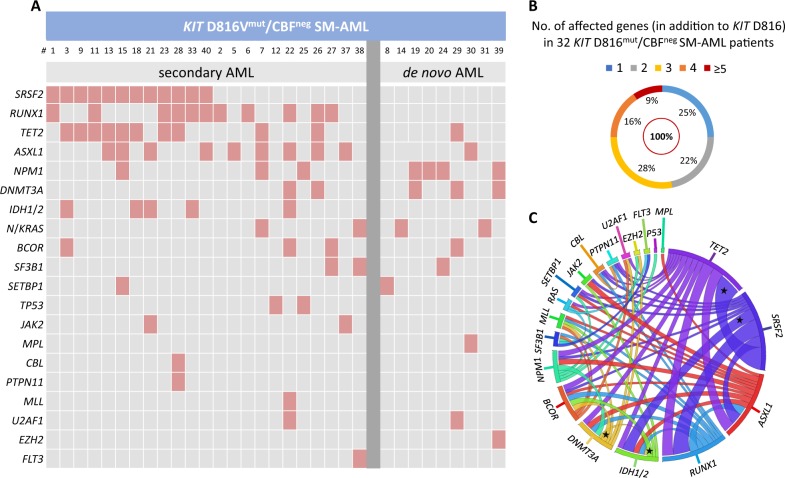
Mutational profile of 32 patients with *KIT* D816^mut^/CBF^neg^ systemic mastocytosis associated with acute myeloid leukemia (SM-AML). **a** Alignment of gene mutations in 32 patients with SM-AML. Each column represents an individual patient, **b** distribution of number of affected genes, and **c** the co-occurrence and overall frequency of mutated genes represented by Circos diagram. Asterisk marks a significant association between several pairs of mutations. Supplementary Table 1 provides the variant allele frequency of all mutations

**Fig. 2:**
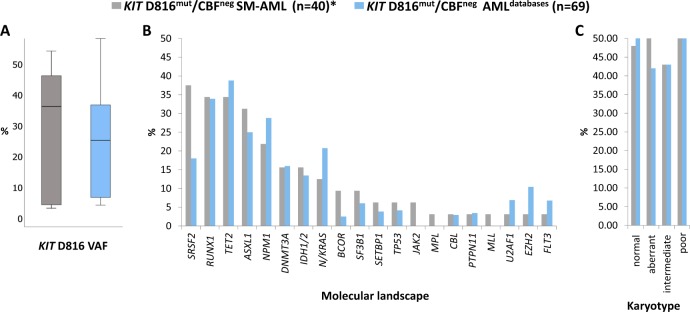
*KIT* D816 variant allele frequency (VAF), somatic mutations, and aberrant karyotype in *KIT* D816^mut^/CBF^neg^ SM-AML in comparison to *KIT* D816^mut^/CBF^neg^ AML from the two databases (AML^databases^). **a** *KIT* D816 VAF, **b** relative frequency distribution of mutated genes, and **c** aberrant karyotype. Gray columns: *KIT* D816^mut^/CBF^neg^ SM-AML and blue columns: *KIT* D816^mut^/CBF^neg^ AML^databases^. Asterisk represents targeted next-generation sequencing was performed in 32/40 SM-AML patients

All patients were positive for *KIT* D816V with a median VAF of 36% (range 3–54). At the time of SM-AML, material for NGS analysis was available from 32/40 (80%) patients (Fig. 1a, Supplementary Table 1). All 32 patients had at least one additional somatic mutation (median 3, range 1–6) and 24/32 (75%) patients had ≥2 somatic mutations in addition to *KIT* D816V (Fig. 1b). There was a significant association between several pairs of mutations, specifically *TET2*/*SRSF2, IDH1*/*2*/*SRSF2, IDH1*/*2*/*BCOR*, and *DNAMT3A*/*BCOR* (*P* < 0.05) (Fig. 1c). The most frequently mutated genes were *SRSF2* (*n* = 12, 38% of patients), *RUNX1* (*n* = 11, 34%), *TET2* (*n* = 11, 34%), *ASXL1* (*n* = 10, 31%), *NPM1* (*n* = 7, 22%), *DNMT3A* (*n* = 5, 16%), *IDH1*/*2* (*n* = 5, 16%), *N*/*KRAS* (*n* = 4, 13%), *BCOR* (*n* = 3, 9%), *SF3B1* (*n* = 3, 9%), *SETBP1* (*n* = 2, 6%), *TP53* (*n* = 2, 6%), and *JAK2* (*n* = 2, 6%). *CBL*, *EZH2, FLT3, MLL, MPL*, *PTPN11*, and *U2AF1* were less frequently affected (<5%) (Fig. 2b).

At least one somatic mutation in *SRSF2*, *ASXL1*, and/or *RUNX1* (S/A/R^pos^) was identified in 21/32 (66%) patients. The rate of S/A/R^pos^ patients was significantly higher in secondary AML (20/23, 87%) as compared to de novo AML (1/9, 11%, *P* = 0.0001). Furthermore, there was a significant correlation between S/A/R^pos^ and age >60 years (*P* = 0.02).

To further evaluate whether *KIT* D816^mut^ occurred in hematopoietic progenitor cells, we performed molecular analyses on DNA derived from CD34+ cells from 6 *KIT* D816V positive patients. *KIT* D816V was found in 1/6 (17%) patients while additional somatic mutations were detected in all 6 patients.

### Cytogenetic analyses

At diagnosis of SM-AML, 19/40 (48%) patients had a normal and 21/40 (52%) patients an aberrant karyotype. All patients were CBF^neg^. Intermediate-risk and poor-risk AML karyotype were diagnosed in 7/21 (33%) and 14/21 (67%) patients, respectively (Fig. 2c, Supplementary Table 2) [27].

### Longitudinal molecular and cytogenetic analyses in patients with secondary SM-AML

In 16/29 (55%) patients with secondary SM-AML, material from the time of diagnosis of SM ± AHN and from the time of diagnosis of secondary SM-AML was available for molecular and cytogenetic analyses. At the time of SM ± AHN, 11/16 (69%) patients were S/A/R^pos^ (Table 2). Acquisition of new somatic mutations and/or karyotype evolution at the time of secondary SM-AML was observed in 15/16 (94%) patients: 4 patients revealed acquisition of new somatic mutations (*NPM1*, *n* = 2; *IDH2*, *n* = 1; *JAK2*, *n* = 1) without karyotype evolution, 5 patients with karyotype evolution, and 6 patients with acquisition of new somatic mutations (*TP53*, *n* = 2; *NPM1*, *n* = 1; *RUNX1*, *n* = 1; *ASXL1*, *n* = 1; *BCOR*, *n* = 1; *IDH1/2*, *n* = 1) and karyotype evolution (Table 2, Supplementary Table 3).

**Table 2:** Longitudinal genetic profile of 16 KIT D816^mut^/CBF^neg^ systemic mastocytosis associated with acute myeloid leukemia (SM-AML) patients who progressed from SM with or without and associated hematologic neoplasm (SM ± AHN)

*MDS myelodysplastic syndrome, MDS/MPN-u* myelodysplastic/myeloproliferative neoplasm unclassifiable, *CMML* chronic myelomonocytic leukemia, *ISM* indolent SM, *MPN-eo* MPN associated with eosinophilia

Boxes highlighted in orange and blue indicate new molecular, karyotype aberrations, respectively

*No karyotype available at the time of SM-AHN

**More (additional) karyotype aberrations

**More (additional) karyotype aberrations

### Treatment modalities and response rate

Thirty-one of 40 (78%) patients were treated with intensive (induction) chemotherapy (*n* = 24, e.g., daunorubicin/cytarabine [DA, 7 + 3], mitoxantrone/cytarabine [S-HAM]) ± non-intensive therapy (hypomethylating agents, *n* = 8, ± cladribine, *n* = 2). The complete response (CR) rates after intensive induction chemotherapy and non-intensive therapy were 40% and 0, respectively. Two patients had cytarabine-based consolidation (without allogeneic stem cell transplantation [SCT]) and are alive 91 and 15 months, retrospectively, after diagnosis of SM-AML. Allogeneic SCT was performed in 12/40 (30%) patients with 4 patients being in CR prior to allogeneic SCT. A durable CR was achieved by 6/12 patients (50%). Nine of 40 (22%) patients received only best supportive care due to advanced age ± comorbidity.

S/A/R^pos^ ± presence of a poor-risk karyotype were negative predictive markers for response to treatment (intensive chemotherapy ± allogeneic SCT) with 10/11 (91%) non-responders presenting with S/A/R^pos^ ± poor-risk karyotype. On the other hand, 4/8 (50%) responders were S/A/R^pos^ ± poor-risk karyotype (*P* = 0.04) indicating that intensive treatment should not be withheld in this subgroup.

### Comparison of *KIT* D816^mut^/CBF^neg^ SM-AML with *KIT* D816^mut^/CBF^neg^ AML from two independent databases

To further investigate whether *KIT* D816^mut^/CBF^neg^ AML represents a distinct subtype which is associated with SM and poor prognosis, two independent AML databases (AML^databases^) were retrospectively screened for *KIT* D816^mut^/CBF^neg^ AML patients. Overall, 69 *KIT* D816^mut^/CBF^neg^ AML^databases^ patients could be identified. Mutation profile and karyotype were available from all patients, detailed clinical characteristics from 17/69 patients (Tables 1b and 3).

**Table 1b:** Clinical characteristics, treatment modalities and outcome of 69 patients with *KIT* D816^mut^/CBF^neg^ acute myeloid leukemia (AML)

*n*	Variables	
	No. of patients (*n*)	69
	Age in years, median (range)	66 (23–86)
	Males, *n* (%)	40 (58)
**69**	**Diagnosis**	
	AML, *n* (%)	50 (72)
	sAML, *n* (%)	19 (28)
**17**	**Treatment modalities**	
	Induction (intensive chemotherapy), *n* (%)	17 (100)
	Consolidation (chemotherapy), *n* (%)	8 (59)
	Consolidation (allogeneic SCT), *n* (%)	7 (41)
**17**	**Outcome**	
	Follow-up in months, median (range)	26 (4–113)
	Deaths, *n* (%)	10 (59)

*n* number, *sAML* secondary AML, *SCT* stem cell transplantation

**Table 3:** Comparison between *KIT* D816^mut^/CBF^neg^ SM-AML and *KIT* D816^mut^/CBF^neg^ AML cases regarding molecular pattern, aberrant karyotype, *KIT* D816 variant allele frequency (VAF), and overall survival (OS)

Variables	*KIT* D816^mut^/CBL^neg^ SM-AML (*n* = 40)	*KIT* D816^mut^/CBL^neg^ AML^a^ (*n* = 69)	*P*-value
*KIT* D816 VAF, median in % (range)	34 (3–54)	29 (3–93)	n.s.
S/A/R^pos^, *n* (%)	21/32 (66)	27/54 (50)	n.s.
FLT3^pos^, *n* (%)	1/32 (3)	4/59 (7)	n.s.
Aberrant karyotype, *n* (%)	21/40 (52)	28/66 (42)	n.s.
OS^b^, median in months (95% CI)	16.7 (9–24)	26.4 (0–61)	n.s.

*n.s.* non-significant, *FLT3*^*pos*^ mutation in FLT3, *S/A/R*^*pos*^ at least one mutation in *SRSF2*, *ASXL1*, and/or *RUNX1*

^a^From the two AML databases (data on OS from 17/69 patients)

^b^Data on patients treated with intensive chemotherapy ± allogeneic stem cell transplantation only

This comparison revealed remarkable molecular and karyotype similarities between the *KIT* D816^mut^/CBF^neg^ SM-AML and the *KIT* D816^mut^/CBF^neg^ AML^databases^ cohort (Fig. 2a–c, Table 3): (a) The median *KIT* D816 VAF was 34% (range 3–54) and 29% (range 3–93), respectively, (b) with the exception of *SRSF2* (38% vs. 18%), the frequency of the most frequently somatic mutations (*RUNX1*, *TET2*, *ASXL1*, *NPM1*, *DNMT3A*, *IDH1*/*2*) was highly similar between the two groups, (c) in contrast to de novo AML, the frequency of *FLT3* aberrations was very low (3% and 7%, respectively), and (d) the frequency of an aberrant karyotype was 52% and 42, respectively, with a comparable rate of intermediate-risk and poor-risk karyotype.

**Fig. 3:**
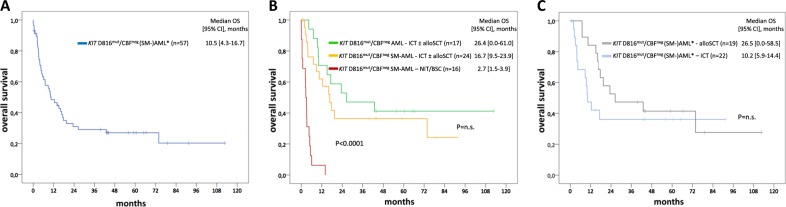
Kaplan–Meier estimates of overall survival (OS) of *KIT* D816^mut^/CBF^neg^ SM-AML and AML from the databases (AML^databases^). **a** OS of all *KIT* D816^mut^/CBF^neg^ patients, **b** OS comparing the *KIT* D816^mut^/CBF^neg^ SM-AML cohort with intensive chemotherapy (ICT) ± allogeneic stem cell transplantation (SCT) (yellow), the *KIT* D816^mut^/CBF^neg^ AML^databases^ cohort with ICT ± allogeneic SCT (green), and the *KIT* D816^mut^/CBF^neg^ SM-AML with non-intensive therapy (NIT)/best supportive care (BSC) (red), **c** OS of all *KIT* D816^mut^/CBF^neg^ patients treated with ICT only (blue) or with allogeneic SCT (gray). CI confidence interval, n.s. non-significant. Asterisk refers to included patients with SM-AML and AML^databases^

The median OS of 40 *KIT* D816^mut^/CBF^neg^ SM-AML and 17 evaluable *KIT* D816^mut^/CBF^neg^ AML^databases^ patients was 5.4 (95% confidence interval, CI [1.7–9.1]) and 26.4 (95% CI [0–61.0]) months (*P* = 0.015), respectively. In the *KIT* D816^mut^/CBF^neg^ SM-AML cohort, 16 patients received non-intensive therapy only, with a median OS of 2.7 months (95% CI [1.5–3.9]), while all 17 *KIT* D816^mut^/CBF^neg^ AML^databases^ patients received intensive chemotherapy. Median OS was not significantly different (16.7 vs. 26.4 months, *P* = 0.4) between *KIT* D816^mut^/CBF^neg^ SM-AML and *KIT* D816^mut^/CBF^neg^ AML^databases^ patients who received intensive chemotherapy (Fig. 3a, b).

In a combined analysis of both cohorts, median OS was not significantly different between intensive chemotherapy (*n* = 22) only vs. intensive chemotherapy followed by allogeneic SCT (*n* = 19), 10.2 months (95% CI [5.9–14.4]) vs. 26.5 months (95% CI [0–58.5]), respectively (*P* = 0.3) (Fig. 3c). With exception of age (patients with allogeneic SCT were younger), no significant differences were observed between the two cohorts regarding clinical and molecular genetic characteristics (Table 4).

**Table 4:** Clinical and genetic data of 41 patients with *KIT* D816^mut^/CBF^neg^ (systemic mastocytosis associated with) acute myeloid leukemia (SM-)AML^a^ treated with intensive chemotherapy (ICT) ± allogeneic stem cell transplantation (SCT)

Variables	ICT *n* = 22	Allogeneic SCT *n* = 19	*P*-value
Age, median (range)	63 (23–79)	56 (23–70)	0.04
SM-AML from SM ± AHN, *n* (%)	9 (41)	9 (47)	n.s.
S/A/R^pos^	9/21 (43)	9/18 (50)	n.s.
Poor-risk karyotype, *n* (%)	5/21 (24)	5/18 (28)	n.s.

*n.s.* non-significant, *S/A/R*^*pos*^ at least one mutation in *SRSF2*, *ASXL1*, and/or *RUNX1*

^a^Included patients with SM-AML and AML from the two databases

**Fig. 4:**
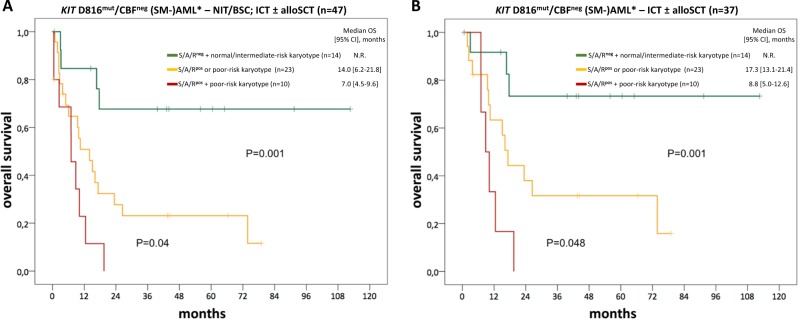
Kaplan–Meier estimates of overall survival (OS) of *KIT* D816^mut^/CBF^neg^ SM-AML and AML^databases^. OS of *KIT* D816^mut^/CBF^neg^ patients treated with **a** non-intensive therapy (NIT)/best supportive care (BSC) or intensive chemotherapy (ICT) ± allogeneic stem cell transplantation (SCT) or **b** ICT ± allogeneic SCT. Depending on the *SRSF2*/*ASXL1*/*RUNX1* (S/A/R) mutation status and karyotype, three different cohorts were identified: S/A/R^neg^ + normal-/intermediate-risk karyotype (green), S/A/R^pos^ or poor-risk karyotype (yellow), and S/A/R^pos^ + poor-risk karyotype (red). CI confidence interval, n.s. non-significant, S/A/R^pos^ at least one mutation in the S/A/R gene panel. Asterisk refers to included patients with SM-AML and AML^databases^

In univariate analyses (including age, hemoglobin, platelets, AML subtype, treatment modalities [non-allogeneic vs. allogeneic SCT], somatic mutations, and aberrant karyotype), only age >60 years, at least one additional somatic mutation in the S/A/R gene panel (S/A/R^pos^) and a poor-risk karyotype were identified as poor prognostic variables regarding OS. In multivariate analysis, S/A/R^pos^ and a poor-risk karyotype remained the only independent adverse factors with regard to OS. Accordingly, a weighted score (based on the HR) of 1 was assigned to S/A/R^pos^ and poor-risk karyotype. Significantly different OS probabilities were observed for the comparisons S/A/R^neg^ + normal-/intermediate-risk karyotype (0 point, *n* = 14), S/A/R^pos^ or poor-risk karyotype (1 point, *n* = 23), and S/A/R^pos^ + poor-risk karyotype (2 points, *n* = 10) with median OS not reached vs. 14.0 [6.2–21.8] vs. 7.0 months [4.5–9.6] (*P* = 0.001). These results were independent of treatment modalities (Fig. 4a, b).

## Discussion

We report here on a large series of 40 patients with morphologically proven *KIT* D816^mut^/CBF^neg^ SM-AML. Approximately 65% of patients evolved from other advSM subtypes. Similar to previous reports concerning the molecular profile of advSM, all patients with SM-AML had at least one additional somatic mutation, most frequently affecting *TET2*, *SRSF2*, *ASXL1*, *RUNX1*, and *NPM1*. In contrast to de novo AML, only one patient had a *FLT3* mutation. The overall molecular profile of SM-AML therefore was more similar to the profile of advSM than to that of de novo AML [28].

Using CFU-GM-colonies and microdissected cells, we have previously shown that mast cells and AHN cells are not only positive for *KIT* D816V but also for additional somatic mutations, indicating that both derive from a common progenitor [12]. However, a significant proportion of colonies were positive for additional somatic mutations but negative for *KIT* D816V [12]. In line with this and other data demonstrating the absence of *KIT* D816V in myeloid blasts of 50% of SM-AML cases [20], we confirmed the absence of *KIT* D816V but the presence of additional somatic mutations in CD34+ cells in 5 of 6 SM-AML cases, indicating that the additional somatic mutations rather than *KIT* D816V are the driving force for progression to secondary AML. In addition, serial molecular genetic analyses revealed the acquisition of new somatic mutations, e.g., in *NPM1*, *IDH1*/*2*, *RUNX1*, with or without karyotype evolution in >90% of patients as further underlying mechanisms for progression to secondary SM-AML. This data is reminiscent of reports on progression in other myeloid neoplasms such as MDS or MDS/MPN, and our previous reports on progression of SM to advSM or progression within advSM subtypes, e.g., to secondary mast cell leukemia, in which somatic mutations in *NPM1*, *IDH2*, or *RUNX1* were also identified as late events and drivers for disease progression [29–35].

SM-AHN is the most common subtype of advSM but the diagnosis is challenging because the mast cell infiltrate may obscure the AHN and vice versa [20, 36–38]. This is particularly true for AML where the morphological but not the histological examination of bone marrow has been established as a standard diagnostic tool. Recently reported data collected from deep targeted sequencing indicated that *KIT* D816 mutations can be identified in 1–6% of patients with various subtypes of myeloid neoplasms, e.g., MDS, MDS/MPN-u, CMML, polycythemia vera, essential thrombocythemia, or myelofibrosis [39–44]. However, many of these cases have not routinely been screened by histopathology for the presence of co-existing SM. Within our registry, all *KIT* D816V^mut^ patients, who had initially been diagnosed as myeloid neoplasms such as CMML, triple-negative MF, and others, in fact fulfilled the WHO-criteria for a diagnosis of SM-AHN.

We therefore sought to investigate the incidence of *KIT* D816^mut^/CBF^neg^ in retrospective screens of two independent AML databases. Rather unexpectedly, 69 patients were identified which revealed remarkable similarities concerning the high *KIT* D816 VAF, the mutation profile and the aberrant karyotype (Table 2), suggesting that the vast majority of these AML^databases^ patients are likely to have SM-AML. Unfortunately, the lack of bone marrow trephine biopsies at initial diagnosis of AML has not allowed a definite re-evaluation of these cases and formal reclassification as SM-AML. However, based on our data, which are in line with previously published results, an underlying or concomitant SM can be diagnosed in most cases of *KIT* D816V^mut^ AML, when the bone marrow is investigated using standard histopathological and molecular studies.

The median OS of the 40 SM-AML patients was 5.4 months and thus even worse as compared to patients with mast cell leukemia, which is defined by the presence of ≥20% mast cells in a bone marrow smear [29]. No patient achieved a CR on treatment with hypomethylating agents and none of the patients was treated with midostaurin. Following intensive induction chemotherapy in eligible patients, the CR rate of 40% was significantly inferior as compared to the general CR rate of de novo AML (70–80%) [45] and median survival following intensive chemotherapy with or without allogeneic SCT was 17 months. In addition to the aforementioned similarities regarding the molecular genetic characteristics (*KIT* D816V VAF, additional somatic mutations, and aberrant karyotype), the poor median OS of 26 months in 17 *KIT* D816^mut^/CBF^neg^ AML patients from the two independent AML databases adds further evidence that *KIT* D816^mut^/CBF^neg^ AML may in fact represent SM-AML in the vast majority, if not all patients. Independently of treatment modalities and consistent with previous reports on other advSM subtypes, e.g., mast cell leukemia, mutations in S/A/R and a poor-risk karyotype conferred an adverse impact on response to treatment, disease progression, and OS [10, 29, 30].

Midostaurin, an orally administered multi-kinase/FLT3-/KIT-inhibitor improves survival in *FLT3*^pos^ AML and achieves overall response rates of 60% in patients with advSM [46, 47]. Better survival is observed in advSM patients without additional somatic mutations in the S/A/R gene panel and a >25% reduction of the *KIT* D816V VAF at month 6 [30, 46–49]. If the presence of SM can be proven in *KIT* D816^mut^/CBF^neg^ AML by bone marrow histology and elevated serum tryptase, KIT inhibitors (e.g., midostaurin, potentially avapritinib [BLU-285, Blueprint Medicines, Cambridge, MA, USA]) in combination with intensive chemotherapy and allogeneic SCT may help to improve the poor prognosis of this distinct AML subtype [50, 51].

We conclude that (a) progression to secondary AML from a preceding *KIT* D816^mut^ SM-AHN is frequently observed and may be triggered by the acquisition of additional somatic mutations with or without karyotype evolution, (b) *KIT* D816^mut^/CBF^neg^ AML is a distinct subtype with remarkable similarities compared to SM-AML cases concerning *KIT* D816 VAF mutation profile, aberrant karyotype, and poor prognosis, suggesting that a significant proportion of these AML patients may in fact have SM-AML, which is a strong argument to propose a new evaluation, (c) with its very high positive and negative predictive value, serum tryptase is an excellent screening marker for SM and should therefore be part of the diagnostic workflow in all AML patients. Cases with an elevated serum tryptase level should subsequently be screened for *KIT* D816^mut^, and (d) bone marrow histology is mandatory in *KIT* D816^mut^ patients. This simple diagnostic procedure will allow reclassification to SM-AML and thus allow inclusion of KIT inhibitors in established treatment modalities of AML.

## Supplementary information

Supplementary Material
