# Supplementary material for: KIT D816 mutated/CBF-negative acute myeloid leukemia: a poor-risk subtype associated with systemic mastocytosis
Source: Leukemia. 2019 Jan 11;33(5):1124–34. doi: 10.1038/s41375-018-0346-z (PMC6756067; doi:10.1038/s41375-018-0346-z)
Supplement: Supplementary file 1 — Supplementary Material [file 41375_2018_346_MOESM1_ESM.docx]

Table 1: Mutational profile of 32 patients with *KIT* D816^mut^/CBF^neg^ systemic mastocytosis associated with acute myeloid leukemia (SM-AML). (A) Alignment of gene mutations in 32 patients with SM-AML including the variant allele frequency (%). Each column represents an individual patient.

|  | ***KIT* D816^mut^/CBF^neg^ SM-AML** | | | | | | | | | | | | | | | | | | | | | | | | | | | | | | | |
| --- | --- | --- | --- | --- | --- | --- | --- | --- | --- | --- | --- | --- | --- | --- | --- | --- | --- | --- | --- | --- | --- | --- | --- | --- | --- | --- | --- | --- | --- | --- | --- | --- |
|  | **secondary AML** | | | | | | | | | | | | | | | | | | | | | | | ***de novo* AML** | | | | | | | | |
| ***#*** | **1** | **3** | **9** | **11** | **13** | **15** | **18** | **21** | **23** | **28** | **33** | **40** | **2** | **5** | **6** | **7** | **12** | **22** | **25** | **26** | **27** | **37** | **38** | **8** | **14** | **19** | **20** | **24** | **29** | **30** | **31** | **39** |
| ***KIT D816V*** | 2 | 1 | 3 | 1 | 0 | 42 | 41 | 14 | 21 | 2 | 47 | 1 | 44 | 3 | 2 | 24 | 1 | 3 | 49 | 45 | 2 | 40 | 1 | 21 | 1 | 54 | 2 | 43 | 3 | 1 | 28 | 2 |
| ***SRSF2*** | 52 | 23 | 34 | 41 | 47 | 45 | 39 | 50 | 29 | 55 | 35 | 35 |  |  |  |  |  |  |  |  |  |  |  |  |  |  |  |  |  | 4 |  |  |
| ***RUNX1*** | 86 |  |  | 99 |  |  |  |  | 36 | 28 | 74 | 80 | 19 |  | 28 |  |  | 39 |  | 46  22 | 40 |  |  |  |  |  |  |  |  |  |  |  |
| ***TET2*** |  | 66 | 42  44 | 46  48  49 | 51 | 46 | 47  53 |  | 42 | 46  51 |  |  |  |  |  | 48  37  51 |  |  |  | 47 |  |  |  |  |  |  |  |  | 42 |  |  |  |
| ***ASXL1*** |  |  |  |  | 36 |  |  | 15  7 |  |  |  | 29 |  | 10 |  | 36 |  | 41 |  | 42 |  | 45 |  |  |  |  |  |  |  | 5 |  |  |
| ***NPM1*** |  |  |  |  |  | 28 |  |  |  |  |  |  |  |  |  | 32 |  |  | 28 |  |  |  |  |  |  | 43 | 25 | 42 |  |  |  | 40 |
| ***DNMT3A*** |  |  |  |  |  |  |  |  |  |  |  |  |  |  |  |  |  | 36 |  | 42 |  |  |  |  |  | 49 |  |  | 42 |  |  | 27 |
| ***IDH1*/*2*** |  | 25 |  |  |  |  | 44 | 57 |  |  | 43 |  |  |  |  |  |  | 38 |  |  |  |  |  |  |  |  |  |  |  |  |  |  |
| ***N/KRAS*** |  |  |  |  |  |  |  |  |  |  |  |  |  |  |  | 36 |  |  |  |  |  |  | 15 |  | 55 |  |  |  |  | 3 | 7 |  |
| ***BCOR*** |  | 38 |  |  |  |  |  |  |  |  |  |  |  |  |  |  |  | 3  81 |  |  | 8 |  |  |  |  |  |  |  | 39 |  |  |  |
| ***SF3B1*** |  |  |  |  |  |  |  |  |  |  |  |  |  |  |  |  |  |  |  |  | 39 |  | 35 | 43 |  |  |  | 48 |  |  |  |  |
| ***SETBP1*** |  |  |  |  |  | 5 |  |  |  |  |  |  |  |  |  |  |  |  |  |  |  |  |  |  |  |  |  |  |  |  |  |  |
| ***TP53*** |  |  |  |  |  |  |  |  |  |  |  |  |  |  |  |  | 98 |  | 63 |  |  |  |  |  |  |  |  |  |  |  |  |  |
| ***JAK2*** |  |  |  |  |  |  |  | 12 |  |  |  |  |  |  |  |  |  |  |  |  |  | 5 |  |  |  |  |  |  |  |  |  |  |
| ***MPL*** |  |  |  |  |  |  |  |  |  |  |  |  |  |  |  |  |  |  |  |  |  |  |  |  |  |  |  |  |  | 5 |  |  |
| ***CBL*** |  |  |  |  |  |  |  |  |  | 52 |  |  |  |  |  |  |  |  |  |  |  |  |  |  |  |  |  |  |  |  |  |  |
| ***PTPN11*** |  |  |  |  |  |  |  |  |  | 39 |  |  |  |  |  |  |  |  |  |  |  |  |  |  |  |  |  |  |  |  |  |  |
| ***MLL*** |  |  |  |  |  |  |  |  |  |  |  |  |  |  |  |  |  |  |  |  |  |  |  |  |  |  |  |  |  |  |  |  |
| ***U2AF1*** |  |  |  |  |  |  |  |  |  |  |  |  |  |  |  |  |  |  |  |  |  |  |  |  |  |  |  |  | 42  41 |  |  |  |
| ***EZH2*** |  |  |  |  |  |  |  |  |  |  |  |  |  |  |  |  |  |  |  |  |  |  |  |  |  |  |  |  |  |  |  | 3 |
| ***FLT3*** |  |  |  |  |  |  |  |  |  |  |  |  |  |  |  |  |  |  |  |  |  |  | 50 |  |  |  |  |  |  |  |  |  |

Table 2: Karyotype aberrations in 40 *KIT* D816^mut^/CBF^neg^ systemic mastocytosis associated with acute myeloid leukemia (SM-AML) patients.

| **Case #** | **Karyotype at diagnosis of SM-AML** | **Risk stratification (ELN 2017 recommendation for AML)^49^** |
| --- | --- | --- |
| 1 | 48,XY,+8,+13[2]  49,idem,+21[5]  52,idem,+9,+18,+22[1]  46,XY[17] | poor |
| 2 | 46,XY,del(5q)[25] | poor |
| 3 | 46,XY,+8[19] | intermediate |
| 4 | 46,XX[20] | favorable |
| 5 | 46,XY,t(16;17)(p11;q11)[18] | intermediate |
| 6 | 47,XY,+r(17)(p11q11)[12]  46,XY,der(10)t(10;15)(p12;q25),der(12)t(12;17)(p11;q23)  del(15)(q21),der(17)t(15;17)(q21;q25) t(15;10)(q25;p12)[2]  46,XY[5] | poor |
| 7 | 47,XX,t(5;10)(q34;p15),+8,der(19)t(3;19)(q11;q13)[7]  47,XX,+8,der(19)t(3;19)(q11;q13)[11]  47,XX,+8[1] | poor |
| 8 | 45,XY,-7[20] | poor |
| 9 | 46,X,idic()x)(q12)del(X)(p11)[3]  47,X,idic(X)(q12),+idic(X)(q12)[2]  46,XX[3] | poor |
| 10 | 44,XY,del(5)(q14q34),del(7)(q21q35),dic(12;13)(p12;p11)  der(17)t(1;17)(p22;p13),der(17;22)(q10;q10)[6]  43,XY,del(5)(q14q34),-7,+r(8)(q24q23),der(8;15)(q10;q10), dic(12;16)(p11;q11),der(17;22)(q10;q10)[5]  46,XY[2] | poor |
| 11 | 46,XY[20] | favorable |
| 12 | 46,XX,del(5)(q14q34)[1]  44,XX,der(3)t(3;13)(p14;p13),del(5)(q14q34),del(7)(q22q36),  r(11)(p15q23)hsr(11)(q23)13,der(17;21)(q10;q10)del(17)(q11q21)[14]  46,XX[5] | poor |
| 13 | 46,XY,+9[20] | intermediate |
| 14 | 46,XX,del(8)(q21),der(10)t(8;10)(q22;q26),  r(12)(p12q15),der(16)t(12;16)(q21;q22),  inv(12)(q21q24)[8]  46,XX[13] | poor |
| 15 | 46,XY[19] | favorable |
| 16 | 46,XY[20] | favorable |
| 18 | 46,XY[20] | favorable |
| 20 | 46,XY[18] | favorable |
| 21 | 46,XX[16] | favorable |
| 22 | 46,XX[20] | favorable |
| 23 | 46,XY[16] | favorable |
| 24 | 46,XY[20] | favorable |
| 26 | 45,XY,-7[20] | poor |
| 27 | 46,XY[20] | favorable |
| 29 | 46,XX,del(17)[19] | poor |
| 30 | 45,XY,-7[20] | poor |
| 31 | 46 XY,+8[8]  46,XY[2] | intermediate |
| 34 | 46,XY[22] | favorable |
| 35 | 46,XY[24] | favorable |
| 36 | 49,XY,+13,+19,+21[14]  46,XY[6] | poor |
| 38 | 46,XX[20] | favorable |
| 39 | 46,XY,t(2;2)[8]  47,XY,t(2;2),+8[5]  46,XY[6] | poor |
| 40 | 46,XY,t(12;22) | intermediate |
| 41 | 46,XY[20] | favorable |
| 43 | 46,XX[22] | favorable |
| 44 | 47,XY,+8[10]  46,XY[10] | intermediate |
| 45 | 46,XX[18] | favorable |
| 46 | 47,XY,+8,del(12)(p12p13)[17]  46,XY[3] | intermediate |
| 47 | 46,XY[20] | favorable |
| 48 | 46,XY[20] | favorable |

Table 3: Longitudinal genetic profile of 16 *KIT* D816^mut^/CBF^neg^ systemic mastocytosis associated with acute myeloid leukemia (SM-AML) patients progressed from SM with or without and associated hematologic neoplasm (SM±AHN).

| **Case #** | **Diagnosis**  **(SM with associated)** | **Time to progression**  **(months)** | **Karyotype**  **(initial and at progression)** |
| --- | --- | --- | --- |
| 15 | MPN-eo |  | 46,XY[20] |
|  | AML | 13 | 46,XY[19] |
| 20 | CMML |  | 46,XY[20] |
|  | AML | 19 | 46,XY[18] |
| 23 | MDS/MPN-u |  | 46,XY[18] |
|  | AML | 37 | 46,XY[16] |
| 38 | ASM |  | 46,XX[20] |
|  | AML | 110 | 46,XX[20] |
| 39 | MDS/MPN-u |  | 46,XY[19] |
|  | AML | 8 | 46,XY,t(2;2)[8]  47,XY,t(2;2),+8[5]  46,XY[6] |
| 9 | CMML |  | 46,XX[20] |
|  | AML | 14 | 46,X,idic()x)(q12)del(X)(p11)[3]  47,X,idic(X)(q12),+idic(X)(q12)[2]  46,XX[3] |
| 5 | ISM |  | 46,XY[22] |
|  | AML | 22 | 46,XY,t(16;17)(p11;q11)[18] |
| 1 | CMML |  | 46,XY[21] |
|  | AML | 29 | 48,XY,+8,+13[2]  49,idem,+21[5]  52,idem,+9,+18,+22[1]  46,XY[17] |
| 2 | CMML |  | - |
|  | AML | 5 | 46,XY,del(5q)[25] |
| 3 | MDS/MPN-u |  | 46,XY[20] |
|  | AML | 4 | 46,XY,+8[19] |
| 6 | ISM |  | 46,XY[17] |
|  | AML | 22 | 47,XY,+r(17)(p11q11)[12]  46,XY,der(10)t(10;15)(p12;q25),der(12)t(12;17)(p11;q23)  del(15)(q21),der(17)t(15;17)(q21;q25) t(15;10)(q25;p12)[2]  46,XY[5] |
| 29 | ISM |  | 46,XX[21] |
|  | AML | 74 | 46,XX,del(17)[19] |
| 7 | CMML |  | - |
|  | AML | 41 | 47,XX,t(5;10)(q34;p15),+8,der(19)t(3;19)(q11;q13)[7]  47,XX,+8,der(19)t(3;19)(q11;q13)[11]  47,XX,+8[1] |
| 36 | MPN-eo |  | 47,XY,19+[16]  46,XY[5] |
|  | AML | 12 | 49,XY,+13,+19,+21[14]  46,XY[6] |
| 12 | MDS |  | 46,XX,del(5)(q13q31)[20] |
|  | AML | 24 | 46,XX,del(5)(q14q34)[1]  44,XX,der(3)t(3;13)(p14;p13),del(5)(q14q34),del(7)(q22q36),  r(11)(p15q23)hsr(11)(q23)13,der(17;21)(q10;q10)del(17)(q11q21)[14]  46,XX[5] |
| 13 | CMML |  | 46,XY,+9[20] |
|  | AML | 48 | 46,XY,+9[20] |

Abbreviations: MDS/MPN-u, myelodysplastic/myeloproliferative neoplasm unclassifiable; CMML, chronic myelomonocytic leukemia; ISM, indolent SM; MPN-eo, MPN associated with eosinophilia.
